# Peptide Conjugation via CuAAC ‘Click’ Chemistry

**DOI:** 10.3390/molecules181113148

**Published:** 2013-10-24

**Authors:** Abdullah A. H. Ahmad Fuaad, Fazren Azmi, Mariusz Skwarczynski, Istvan Toth

**Affiliations:** 1 School of Chemistry and Molecular Biosciences, University of Queensland, Brisbane, QLD 4072, Australia; E-Mails: abdullah.ahmadfuaad@uqconnect.edu.au (A.A.H.A.F.); fazren.azmi@uqconnect.edu.au (F.A.); mariusz.skwarczynski@uq.edu.au (M.S.); 2 School of Pharmacy, University of Queensland, Woolloongabba, QLD 4012, Australia

**Keywords:** CuAAC, click chemistry, chemical ligation, peptide ligation

## Abstract

The copper (I)-catalyzed alkyne azide 1,3-dipolar cycloaddition (CuAAC) or ‘click’ reaction, is a highly versatile reaction that can be performed under a variety of reaction conditions including various solvents, a wide pH and temperature range, and using different copper sources, with or without additional ligands or reducing agents. This reaction is highly selective and can be performed in the presence of other functional moieties. The flexibility and selectivity has resulted in growing interest in the application of CuAAC in various fields. In this review, we briefly describe the importance of the structural folding of peptides and proteins and how the 1,4-disubstituted triazole product of the CuAAC reaction is a suitable isoster for an amide bond. However the major focus of the review is the application of this reaction to produce peptide conjugates for tagging and targeting purpose, linkers for multifunctional biomacromolecules, and reporter ions for peptide and protein analysis.

## 1. Introduction

Peptides and proteins were discovered in the beginning of the 20th century. However, it took more than 50 years for scientists to understand their natural biosynthesis pathway. In cells, the biosynthesis of peptides and proteins starts with the transcription of deoxyribonucleic acid (DNA) sequences to ribonucleic acid (RNA) sequences, which is also known as messenger RNA (mRNA). These mRNA transcripts are translated into peptides or proteins in the ribosome. In this biological ‘mechanical’ ligation factory within living cells, amino acids are conjugated to one another with the help of transfer RNA (tRNA). The resultant proteins perform crucial roles in living organisms, serving as enzymes, structural proteins, signaling proteins *etc*. The broad spectrum of biological activities has made proteins an attractive component of modern pharmaceutics. For example, insulin, used as a drug to control blood sugar level in patients with Type 1 diabetes, was originally extracted from cows and pigs, purified and used in humans as a therapeutic drug [1]. To improve this process, scientists have tried to mimic the conjugation process *in vitro*. Chemical peptide synthesis began when Theodor Curtius succeeded in conjugating the first N-protected dipeptide, benzoylglycylglycine, in 1881 [2]. Twenty years later, Emil Fisher published an alternative glycylglycine dipeptide synthesis via a hydrolysis pathway [3]. Since then, interest in peptide synthesis grew and with the introduction of temporary protecting groups such as carbobenzoxy (Cbz) by Bergmann and Zerwas in 1931 [4], the synthesis of the first biologically active peptide hormone (oxytocin) was accomplished by Vigneaud *et al*. [5]. Finally, peptide synthesis was streamlined with the introduction of solid support (also called solid phase peptide synthesis or SPPS) by Merrifield in the early 1960s [4,6]. The SPPS approach allowed the synthesis of complex, chemically synthesized biological active peptides such as human insulin and ribonuclease A enzyme [4,7]. Peptides up to 50 amino acids in length can be efficiently synthesized by SPPS in a relatively short time. Major drawbacks of SPPS were observed for the synthesis of longer peptide (>50 amino acids) where the solubility of the growing peptide and accumulation of by-products on the solid support resulted in poor purity and yield [6].

New chemical synthesis techniques were then developed to combine two or more peptide fragments to form a longer construct. For example, Kimura *et al*. in 1981 used a segment condensation reaction technique where 13 fragments of five amino acid long peptides (each with protected side chain) were conjugated together in water to form a functional protein [8]. This reaction is typically limited by epimerization of enantiomerically active residues at the C-terminus of the peptide during carboxyl group ‘activation’ prior to the condensation reaction. Additionally, the presence of protected side chains was required to avoid by-product formation due to the reaction of side chain functional groups. The method was extremely tedious and produced a very poor yield of the final product.

**Scheme 1:**
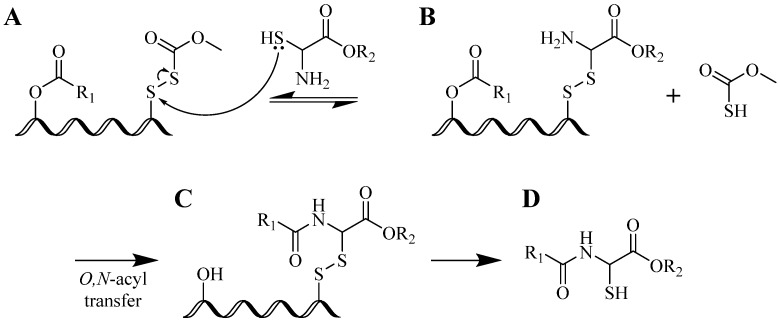
Prior thiol capture involving intramolecular *O*,*N*-acyl transfer reaction. (**A**) Activated thiol species (**B**) captured thiol fragment, and (**C**) after acyl transfer reaction, (**D**) desired peptide is formed.

In the same year, a “prior thiol capture” reaction was introduced by Kemp *et al*. The group reported intramolecular *O*,*N*-acyl transfer with disulfide interchange to transfer an adjacent acetyl group to an amine group at the N-terminal of a cysteine peptide residue, illustrated in Scheme 1 [9]. However, the method has its own limitations: new disulfide bond formation is slow and the whole process resulted in the formation of byproducts.

**Scheme 2:**
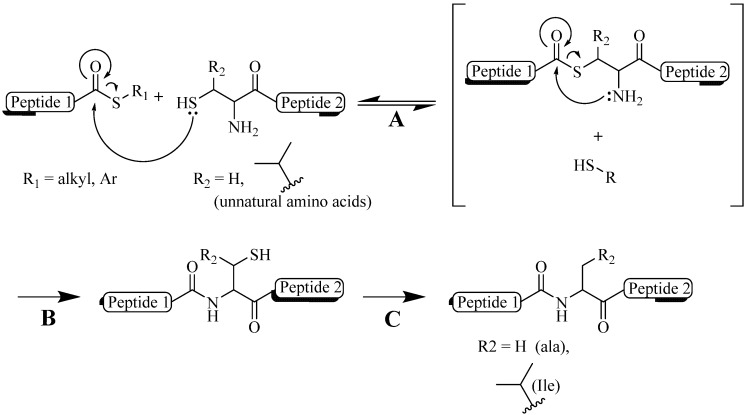
An example of NCL reaction of peptides containing cysteine or its derivatives. Reaction (**A**) intermolecular thioesterification; (**B**) intramolecular S➔N acyl transfer; (**C**) (optional) desulfurization of cysteine derivatives to cysteine residue.

A more advantageous chemical technique called native chemical ligation (NCL) was introduced by Wieland *et al*. in 1953. However, a practical method was not reported, until Kent and co-workers introduced their developments on the technique in the 1990s [6,10,11]. NCL involves the conjugation of a C-terminal thioester peptide to an N-terminal cysteinyl peptide (Scheme 2). This technique was much more efficient in comparison to the approaches previously mentioned, as protection of peptide side chains was not required and final product was produced in high yield and purity. However, the peptide of interest needed to contain a cysteine residue (or its derivatives) within its native sequence, otherwise additional process of desulfurization is required to remove the ‘unwanted’ sulfur group [11,12]. Consequently, during the desulfurization, other sulfur moieties within the peptide construct, if present, must withstand the desulfurization process or else, the sulfur groups will be removed and will resulted in undesired peptide construct [12]. Although NCL is an exceptionally effective method for the production of large peptides and proteins, there were also difficulties encountered during the ligation of hydrophobic target products [13,14]. Additionally, NCL has also been difficult to apply as a conjugation technique between peptides and non-peptidic molecules such as polymer. Thus alternative methods such as Staudinger ligation, Diels-Alder reaction, strain promoting alkyne-azide cycloaddition (SPAAC) and copper catalyzed alkyne-azide cycloaddition reaction (CuAAC) have been developed for further modification of peptides modification.

This review presents advances in the conjugation of peptides to other biomolecules via CuAAC to form 1,4-disubstituted 1,2,3-triazoles. CuAAC is used as an alternative to NCL, the bioconjugation technique widely used in protein and peptide chemistry. This review briefly described the history of CuAAC and the structural similarities of triazole to amide bond, and later presents most recent application of CuAAC as linkers and amide bond isosteres. Additional reviews in regards to NCL, Staudinger ligation, Diels-Alder reaction and SPAAC reactions were recently appraised by Chandrudu *et al*. [15] and Raibout *et al*. [16], van Berkel *et al*. [17], de Araujo *et al*. [18], and Debets *et al*. [19], respectively.

## 2. Copper (I) Catalyzed Alkyne-azide 1,3-Dipolarcycloaddition (CuAAC)

**Scheme 3:**
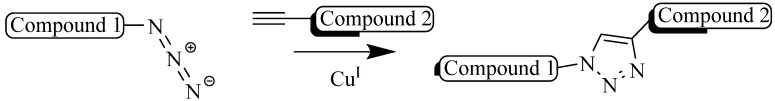
General reaction for CuAAC reaction producing a triazole ring.

The formation of triazole was first discovered and reported by Authur Michael in 1893 [20]. In 1961, Rolf Hüisgen performed systematic studies on the nature of this reaction which was subsequently named the 1,3-dipolar cycloaddition (Scheme 3) [21]. The use of copper for the catalysis of Hüisgen azide-alkyne 1,3-dipolarcycloaddition (CuAAC), was first reported by L’Abbé in 1984 as a side reaction during the synthesis of azidoallenes complex [22]. No further investigation related to this observation was performed until in 2001, when the reaction was introduced by two independent laboratories led by Sharpless in the US and Meldal in Denmark [23,24]. CuAAC reaction, or ‘click’ reaction, is a regioselective copper (I) catalytic reaction between two terminal alkyne and azide functional groups, that give rise to 1,4-disubstituted 1,2,3-triazoles under mild conditions (Scheme 3, detailed possible mechanisms were discussed by Jones *et al*. and Himo *et al*.) [25,26,27,28]. Soon after its discovery, the CuAAC reaction became a common conjugation method, predominantly because this reaction is very robust, selective, and insensitive to the changes in pH and temperature. Currently, CuAAC is used in a wide range of applications in various disciples ranging from biomolecular and medicinal chemistry to polymer sciences [29,30,31].

Although the use of copper (I) is crucial for the cycloaddition reaction, in some cases, regulation of solvent and temperature (by heating in oil bath or microwave irradiation [32,33]), and introduction of ligand molecules or reducing agent can further push the reaction towards its desired product. Many copper (I) sources were tested and reported to catalyze the reaction. These include: copper (I) iodide (CuI), copper (I) bromide (CuBr), copper (II) sulfide (CuSO_4_) or copper (0) (such as copper wire, powder and palette). For example, Meldal and co-workers used CuI and *N*,*N*-diisopropylethylamine (DIPEA, base to pre-activate the Cu^I^ by forming a copper-acetylene complex) in *N*,*N*-dimethylformamide (DMF) at 25 °C to yield 1,4-disubstituted 1,2,3-triazole structures [24]. An alternative method reported by Jang *et al*. involved the introduction of sodium ascorbate (NaAsc, a reducing agent that converted *in situ* copper (II), Cu^II^, into copper (I), Cu^I^) and substitution of DIPEA with pyridine also resulted in formation of the triazole structure [34]. It was reported that removing the base from the reaction mixture usually did not significantly influence the reaction yield, thus base-free CuAAC is often reported [30,35,36]. The optional introduction of a copper ligand helped to enhance the progress of the reaction while protecting the Cu^I^ ions from oxidation. The versatility of this cycloaddition reaction was recently reviewed by Meldal [37].

Nevertheless, the application of CuAAC for biological compounds is controversial in regards to copper toxicity and the use of the reducing agent. For example there were reports that active copper species readily form radicals that can (partially) degrade or destroy peptides and protein complexes during CuAAC reactions, while in *in vitro* system, copper complexes may be taken up by cells, thus altering cellular metabolisms and functions [38,39]. To overcome these limitations, CuAAC-cell compatibility can be improved by either, the use of water-soluble ligands (e.g., bis-(L-histidine) [40]) or, in some cases, the use of accelerating CuI-ligands that allowed low CuI loading during catalytic reaction [41]. It has been also shown that copper wires can catalyze CuAAC reaction without need of the use of any additional ligands or reducing agents [30,42,43,44]. The toxicity of copper is well established; however, at the same time copper is essential element for human health, therefore the level of copper traces presented in the biologically relevant material need to be precisely determined (the recommended health standard level of copper is below 15 ppm) [45].

## 3. Structural Studies of Amide Bond and 1,4-Disubstituted Triazole

**Figure 1:**
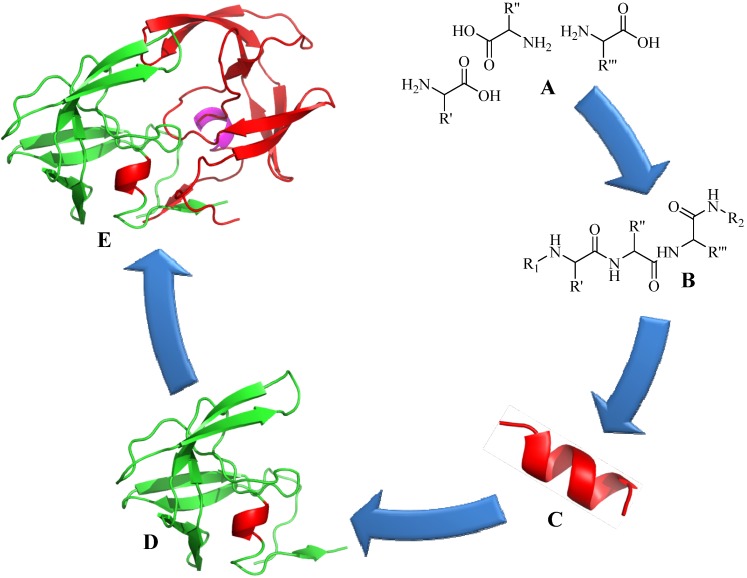
From amino acids to protein. (**A**) free amino acids; (**B**) primary structure (peptide bonds); (**C**) secondary structure (α-helix or β-sheet); (**D**) tertiary structure (whole protein or subdomain protein); (**E**) quaternary structure (multiple domain protein, HIV Protease, Protein Data Bank (PDB) number = 1HSG) [57].

Amide bonds play a very important role in determining the bioactivity of a protein. Amino acids, the building block for proteins, are connected via the amide bonds. These bonds have restricted flexibility which allows distinct protein conformation. This structural conformation is further enhanced by intramolecular interaction between neighboring peptide chains as a result of hydrogen bonding, disulfide bridge formation, or hydrophobic interactions [1]. Turns in the backbone and intramolecular bonding result in proteins adopting a stable conformation (Figure 1). As a result, the incorporation of a single amino acid substitution at any point within the protein may result in altered structure. Thus, when an unnatural element is incorporated into peptide or protein, the ability of the synthetic constructs to mimic the native structure is very important to ensure the synthetic constructs maintain the desired biological activity. For example, it was discovered that single amino acid substitution in a synthetic luteinizing hormone releasing hormone (LHRH) drastically changes the peptide folding thus reduces the its activity [46,47]. Similarly, an antigen in subunit peptide vaccine needs to fold into its native conformation in order for the immune system to be able to recognize it and thus to produce a protective antibodies against the desired pathogen [48].

**Figure 2:**
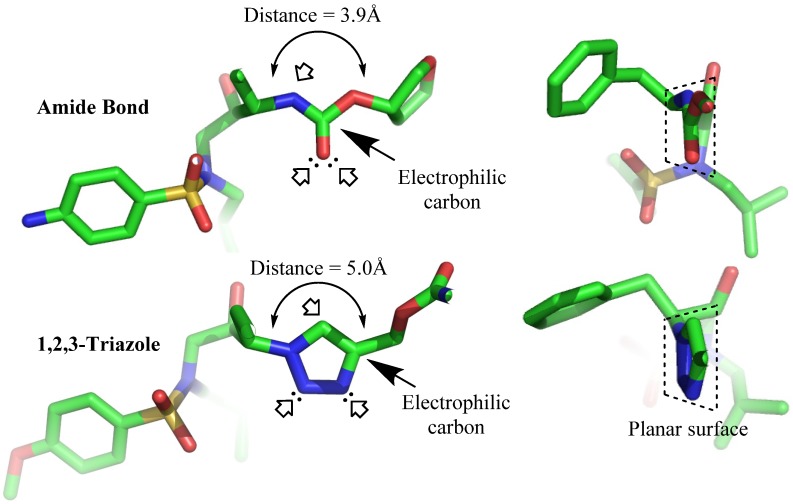
Triazole as amide bond bioisosters. Arrow (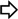 ) represent hydrogen bonding sites. (PDB: 1HPV [59] and 1ZP8 [53]).

The importance of peptide and protein conformation limits the ability to easily substitute peptide bonds with unnatural elements. Peptide bonds can be replaced by mimicking functional groups (e.g., ester) [49,50]; however, not all of them, when incorporated in the sequence, are able to maintain secondary structure of the peptide (e.g., alkene) [51]. These facts prompted special interest in the application of triazole moiety for this purpose. The structural studies of amides and triazoles were first performed by Horne *et al*. in 2004 with the modification of pLI-GCN4 sequences, an α-helix coiled coil structure. They reported that although triazole substitution of the amide bond in a peptide backbone is longer by 1.1 Å (Figure 2), the modified peptide was still able to maintain its helical structure [52]. Later, Brik *et al*. supported the idea that the 1,4-disubstituted triazole ring is suitable as a peptide surrogate or bioisostere [53]. The group experimented with triazole analogs of a peptide-based HIV protease inhibitor and found that the modified constructs maintained nanomolar inhibition activity. Crystallographic analyses of the constructs showed that the analogs were bound to the same enzyme pocket as the parent peptide. This finding further supported the observation that the triazole group displayed similar configuration to amide bond, mimicking trans-amide bond arrangement (Figure 2). Moreover, the triazole structure conferred almost similar polarizing properties to those found in amides, including the positions of hydrogen bonding donor and acceptor, and the similar electrophoretic dipole (5 Debye as compared to 4 Debye in amide bonds) [53]. In addition, the triazole ring is able to align itself with other amide groups via hydrogen bonds, in a similar manner to the alignment of an amide group to other amides in peptide secondary structure. The ring also adapted a three dimensional planar structure similar as an amide bond (Figure 2) [54,55,56]. However, unlike a native amide bond, triazoles are stable against proteolytic amide degradation. In sum, it is profound that structural modification of peptides and proteins using the CuAAC reaction could form an effective structural mimic of native amide bonds. 

Triazoles have been found to be excellent peptide bond substituents mainly due to their ability to increase peptides’ biological stability *in vivo* while maintaining their activity. The natural occurring amide bond is very susceptible to various proteases [58]. Thus, substituting the amide bond with a triazole provides an alternative prospect to increase the bioavailability of the target compound *in vivo*. The other advantage of triazole substitution is related to the CuAAC reaction itself. Unlike peptide coupling reactions, CuAAC is selective towards terminal azide and alkyne functional groups. This reaction can therefore be performed on unprotected peptides containing azide/alkyne groups [43]. Moreover, the CuAAC reaction is easy to perform with an ample range of reaction media and copper sources to choose from. Hence, the reaction condition can be altered to suit the conditions of the conjugation reaction. Despite the versatility of CuAAC and the triazole moiety mimicry of the amide bond, in most cases, CuAAC is primarily used for peptide-to-biomolecular conjugation.

## 4. Application of CuAAC in Peptide Modifications

**Figure 3:**
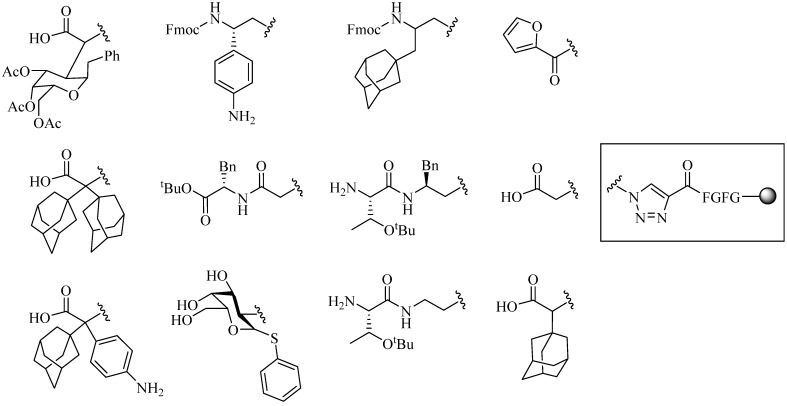
Examples of resin-bound peptidotriazoles constructs synthesized via CuAAC.

In 2002, the first application of the CuAAC reaction to form peptide derivatives was reported with the synthesis of peptidotriazoles and neoglycopeptide-linked-triazoles on solid support by Tornoe *et al*. [24]. A wide variety of azido groups were tested, affording compounds with crude purities ranging from 75% to 99% (Figure 3) [24]. The application of this reaction is not limited to conjugation between molecules (intermolecular coupling), but also within molecules (intramolecular coupling), thus the number of publications that have used this method have grown exponentially. Both inter and intramolecular conjugations have recently been added to the vast number of applications for this technique.

### 4.1. Intermolecular Linker

#### 4.1.1. Single-Site Intermolecular Linker

One of the purposes of azide-alkyne single scaffold conjugation is tagging biomolecules. By labeling the peptides or protein with radioactive molecules (such as iodine-125 or fluorine-18 and their derivatives) or fluorescent compounds (green fluorescent protein, GFP), the target of interest can be visualized via positron emission tomography (PET) or fluorescent imaging (fluorescent microscopy), respectively [60,61,62,63,64,65].

One example for such radiolabeled conjugation was to tag the tumour-targeting high molecular weight peptide, “pH (low) insertion peptide” (pHLIP) with radioactive ^18^F. Direct labeling of large peptide using ^18^F was synthetically challenging due to:

(1) Short half-life (low stability) of ^18^F—thus the synthesis needed to be completed within a short time;
(2) Consideration of the safety of the operator working on the high gamma energy ^18^F;
(3) Low purity resulting from direct coupling of ^18^F, while the use of ^18^F derivatives often required longer and more complicated synthetic procedures [66,67,68].

Although direct ^18^F labeling was possible [65], however, the functional group in the peptide is barely compatible. Thus, CuAAC approach provides the best alternative for such conjugation to occur. Sutcliffe and co-workers successfully synthesized a 20 amino acids α_v_β_6_^18^F-radiolabeled peptide (medium MW ~2,000 Da, 10% yield) via the CuAAC reaction. However, the group experienced major difficulties when trying to conjugate the radiolabelled group to larger peptides via CuAAc [61,63]. A method to overcome this problem was reported by Daumar *et al*. who synthesized and conjugated a novel ^18^F derivative to the pHLIP peptide via CuAAC reaction [61]. The initial CuAAC reaction between the ^18^F-polyethylene glycol (PEG)-alkyne and pHLIP-azido groups under standard conditions (with copper (II) acetate, NaAsc in H_2_O/MeCN (1:1 mixture) at 70 °C) was unsuccessful, despite the fact a similar reaction between ^18^F-PEG-alkyne and RGD-azide peptide (MW ≈ 2,000 Da) proceeded smoothly [61,69]. Radiochemically active ^18^F-pHLIP was obtained using a novel ^18^F-alkyne prosthetic group (^18^Fluoro-pyridine alkyne) under standard conjugation conditions (13% pure yield within 85 min of preparation time) [61]. The use of less polar solvent mixture, *i.e*., ethanol in H_2_O (1:1), was preferable for the CuAAC reaction of the ^18^F derivative and pHLIP as compared to in H_2_O/MeCN (1:1) mixture [61].

**Figure 4:**
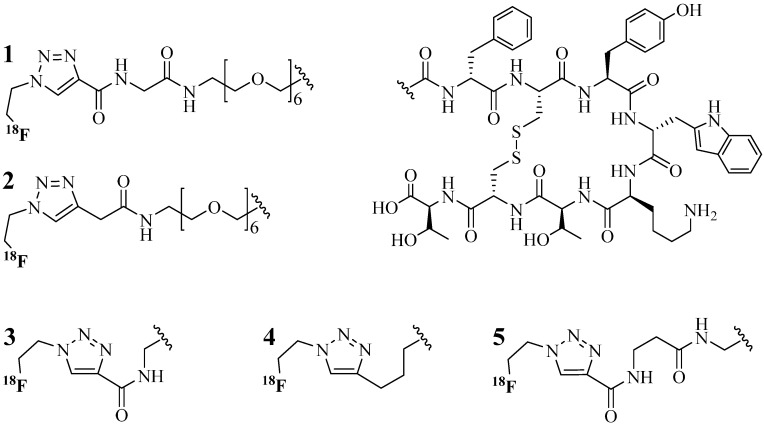
^18^F-labeled TOCA analogs for tumor imaging. CuAAC condition: pH 5 acetate buffer, DMF and acetonitrile (MeCN) (8:3:10) at 25 °C plus; CuSO_4_ and NaAsc.

Octreotide is a peptide that has high receptor specificity to somatostatin receptors overexpressed in neuroendocrine tumours. The Reubi’s group was the first to investigate somatostatin reception via a radiolabeled octreotide analog, [Tyr^3^]octreotate (TOCA) in 1985. The labeling was performed by iodination reaction at the tyrosine (Tyr^3^) side chain [65]. Conversely, the CuAAC reaction was used by Aboagye and colleague to conjugate a different radioactive compound (^18^F) to the TOCA analog (Figure 4) [63]. Aboagye’s group was the first to illustrate the application of CuAAC for tagging molecules through the conjugation of octreotide and a radioactive ^18^F compound (instead of direct labeling of an amino acid within the construct). With the primary aim of reducing the synthesis time and increase TOCA binding affinity, five compounds were prepared using different alkynes analogs to create a novel library of radiolabeled TOCA analogs. The CuAAC synthesis using CuSO_4_ (2 eq.) and NaAsc (2.2 eq.) at pH 5.0 at room temperature resulted in over 98% yield for two out of five compounds (**3** and **5**). Optimizing the reaction conditions by increasing the amount of CuSO_4_ to 4 eq. and NaAsc to 4.4 eq. resulted in excellent yield (>98%) for the other three compounds (**1**, **2** and **4**). The group suggested that the position of the alkyne group located next to the amide group (**3** and **5**) enhanced the kinetics of the CuAAC reaction, while sequential glycol groups near the alkyne moiety reduced the CuAAC reaction rate (**1**) [63]. More detailed study into the influence of functional groups on the CuAAC reaction was reported by Golas *et al*. [70] and Fokin and Hein [71]. Affinity binding experiments with Reubi’s compounds illustrated high binding affinity (IC_50_ < 10 nM) compared to control octreotide peptide (IC_50_ = 15 nM) [63]. This indicated high specificity of the compounds for the somatostatin receptor (CuAAC reaction did not cause loss of binding capacity).

Sewald and colleagues used arginine-aspartic acid-glycine (RDG) peptide as a targeting moiety for anticancer drug delivery [72]. The CuAAC reaction was used to conjugate cyclic RDG peptide to cryptophycins, an apoptosis promoting and tubulin inhibitor depsipeptides (anticancer drug). Unfortunately, conjugation of RDG to cryptophycins reduces the drug’s efficacy. Addition of fluorescein derivative to the drug further decreased its affinity to the microtubule in cancer cells due to steric hindrance. However, confocal analysis of the fluorescein-labeled constructs found that the presence of the cyclic peptide correlated with increased endocytosis by tumor cells. This illustrates the possibility to use the RDG peptide as a tumor-targeting moiety in peptide-drug conjugates. The CuAAC reaction was performed using copper (0) powder in *tert*-butanol/H_2_O (2:1) mixture at room temperature. Copper powder was selected because solid copper is easily removed by filtration. The product yield was moderate with 68% and 43% yield for the non-fluorescent and fluorescently-labeled compounds, respectively, possibly due to short reaction time (~8 h) and the use of copper solid without a ligand [72].

**Figure 5:**
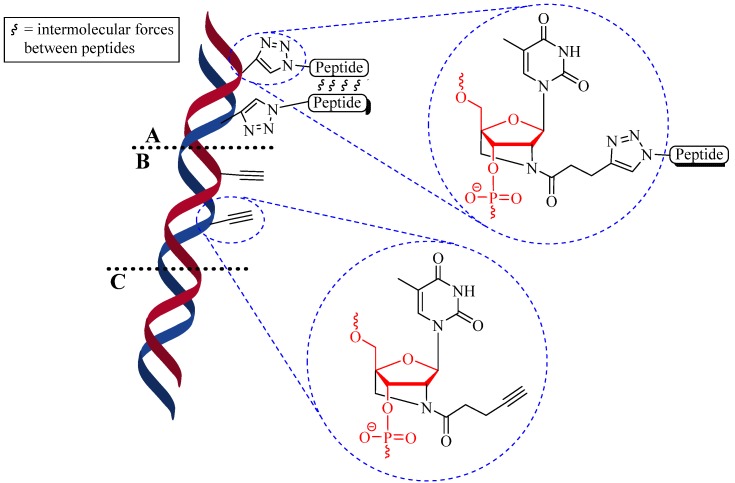
Structural comparison between; (**A**) CuAAC locked-DNA; (**B**) locked-DNA; and (**C**) unmodified DNA. Red structures highlight the DNA backbone.

Besides tagging, the CuAAC reaction was also used to modify the biological properties of peptide-oligonucleotide conjugates (POCs). Astakhova *et al*. conjugated enkephalin peptides to oligonucleotide (deoxyribonucleic acid, DNA) via CuAAC reaction to form POCs and the structure and properties of the oligonucleotides were examined [29]. The POCs were synthesized using CuSO_4_ in the presence of tri(benzyltriazolylmethyl)amine (TBTA) ligand (1:1), NaAsc, aminoguanidine hydrochloride, dimethylsulfoxide (DMSO), 0.2 M carbonate buffer at pH 8.5, under argon atmosphere with vortexing for 12 to 24 h at room temperature [29]. The conjugation yields were over 95% and the method was highly reproducible. Thermal denaturing temperature (T_m_) analysis showed the POCs remained stable at higher temperatures (up to 10 °C higher) than free oligonucleotides. Structural analysis of double POC conjugates resulted in structural stability of the POCs for up to 8 h in diluted human serum (90%) in comparison to a locked-nucleic acid DNA (locked-DNA) and unmodified DNA (control), which were degraded within 1 h and 30 min, respectively (Figure 5) [29].

Furthermore, to improve the efficiency of fluorinated organophosphorous inhibitor (floronated OPI) targeted against serine hydrolases, Sokolova *et al*. constructed a library of small peptide analogs conjugated to the inhibitor via CuAAC reaction (Table 1) [73,74]. Preliminary reactions were performed with CuSO_4_ and NaAsc in chloroform (CHCl_3_)/H_2_O at 10:1 mixture for 1 h at 40 °C. However, the yields were relatively modest (30%–65%) [74]. Prolonged reaction time (3 h) in 1:1 CHCl_3_/H_2_O mixture improved the overall yield to over 75% [73]. Significant inhibition of the fluorinated OPI-peptide conjugates was observed (IC_50_ in milimolar range) with selective inhibition towards BChE and CaE subtype serine hydrolases [74].

**Table 1:** Selected fluoronated OPI-peptide constructs linked via CuAAC reaction. 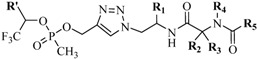

Entry	R′	R1	R2	R3	R4	R5	Yield (%)
**1**	-CF_3_	-CH_3_	-CH_3_	-CH_3_	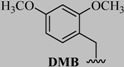	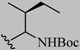	56
**2**	-CF_3_	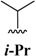	-H	-H	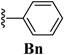	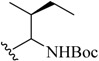	40
**3**	-CF_3_	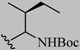	-H	-H	Bn	-CF_3_	45
**4**	-CF_3_	-H	-CH_3_	-CH_3_	Bn	-CF_3_	45
**5**	-C(O)OCH_3_	i-Pr	-H	-H	Bn	-CF_3_	65
**6**	-C(O)OCH_3_	i-Pr	-H	-H	Bn	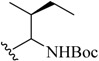	55

Aggregation of elongated peptide during SPPS is often associated with poor yield and purity of the product [75]. With the aim to study the aggregation property of peptides, Perrier and colleagues worked with Alzheimer’s disease-associated β-amyloid peptide (Aβ), which is known for its extreme aggregation, as a model peptide. The group used a microwave-assisted CuAAC reaction to conjugate short Aβ fibrils (sequence 16–20, FVLKFF) to different amphiphilic polymers: polar poly(hydroxylethyl acrylate) (PHEA_20_), and less polar poly(*N*-isopropyl acrylamide) (PNIPAAM_20_) [76]. Microwave-assisted CuAAC was chosen to destabilized peptide aggregation and further enhanced the CuAAC reaction. The reactions were performed using CuSO_4_, NaAsc and DMF at 100 °C for 15 min. After completion of the conjugation, excess alkyne was removed by a secondary CuAAC reaction with the resin-bound azide. The final pure peptide-polymer conjugates were obtained in 43% yield. TEM images illustrated different structural assemblies which were highly dependent on aggregation time and concentration of the CuAAC products [76]. Attachment of the polymers generally disturbs the Aβ aggregation. For example, in the case of PHEA_20_-Aβ conjugates, an 80% reduction in β-sheet formation was observed [76].

**Figure 6:**
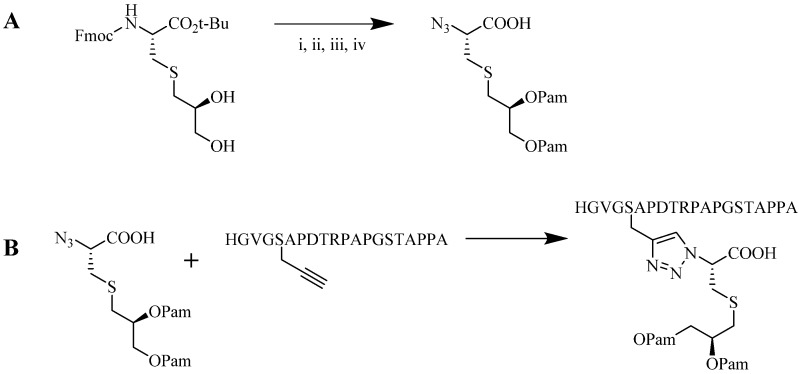
(**A**) synthesis of azide modified Pam_2_Cys via four steps: (i) piperidine, dichloromethane; (ii) imidazole-1-sulfonyl azide, potassium carbonate, methanol; (iii) palmatic acid (Pam), diisopropylcarboiimide, dimethylaminopyridine, tetrahydrofuran; (iv) trifluoroacetic acid; (**B**) CuAAC reaction of Pam_2_Cys construct.

More recently, Brimble and colleagues synthesized novel Pam_2_Cys constructs by conjugating Pam_2_Cys to MUC1 peptide via CuAAC reaction (Figure 6) [77]. The CuAAC reaction was performed using CuI/ triethylphosphine (P(OEt)_3_)/DIPEA in DMF for 30 min at room temperature [77].

#### 4.1.2. Multiple-Sites Intermolecular Conjugates

The CuAAC reaction allows single site conjugation between two molecules. However, multiple molecules can also be specifically conjugated to a single multi-site entity, and two molecules can be associated together using multiple conjugations.

**Figure 7:**
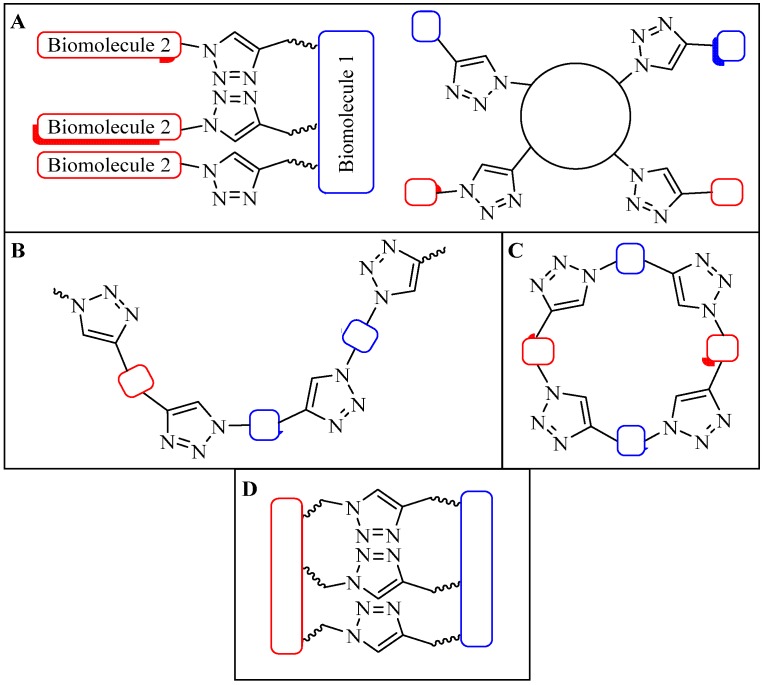
Multiple conjugation strategy using CuAAC approach; (**A**) dendritic, (**B**) linear, (**C**) cyclic, (**D**) cross-linked.

Multiple biomolecule conjugation can be sub-categorized into dendritic, linear, cyclic or cross-linked assemblies (Figure 7). Although these conjugations are typically used in medicinal chemistry, dendrimer and linear assemblies have also been used in the field of vaccine development to enable antigen incorporation in a multiple antigen presenting (MAP) system. This system was shown to induce better immunological responses than a single antigen presenting system [78]. SPPS technique has been employed to synthesize MAP-based constructs. However, the peptides produced are usually difficult to purify to homogeneity. NCL of successive antigens is laborious, and attaching several epitopes at once can be difficult [13,14]. CuAAC provides an alternative to both stepwise SPPS and NCL to efficiently produce such constructs. In contrast to multivalent NCL, CuAAC proceeded faster, resulted in a higher yield, and the triazole product was stable in a biological environment [79].

**Figure 8:**
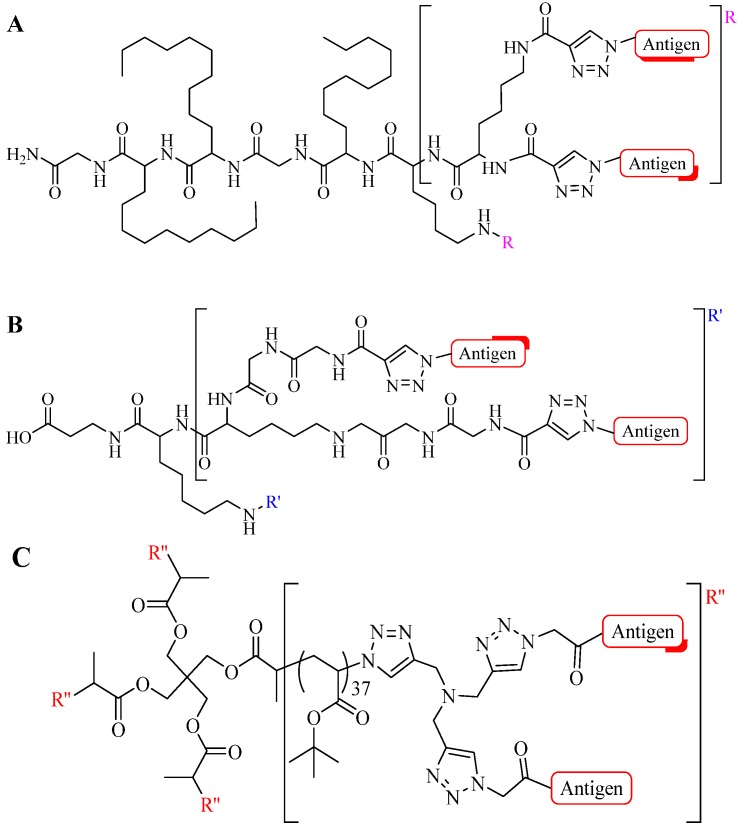
Example of multiple triazole scaffolds synthesized via CuAAC; (**A**) lipid core peptide (LCP), (**B**) dendron scaffold, and (**C**) polymeric dendrimer.

Gupta *et al*. and Skwarczynski *et al*. exploited α- and ε-amino groups in the amino acid lysine as a branching unit for multiple conjugation sites for antigen presentations [43,79,80,81]. Using this technique, a set of azide- or alkyne-modified peptides were selected and conjugated to another peptide core. Conjugation efficiencies above 95% have been reported for CuAAC between functionalized azide and alkyne peptides. Skwarczynski *et al*. performed the CuAAC reaction in DMF, using copper wire as the copper source and heating at 50 °C without additional base, reducing agent or ligand. Within five hours, a 100% conversion was observed (Figure 8A) [43]. An alternative reaction was performed by Gupta *et al*. where CuSO_4_ and NaAsc were used for the CuAAC reaction at room temperature. Quantitative conjugation for a less sterically hindered peptide alkyne was attained in a shorter time (1 h, Figure 8B) [79]. Both constructs were found to be immunologically active in an animal (murine) model, signifying the compatibility of CuAAC with multiple conjugate linkers [43,79,80].

As an alternative, Toth and colleagues conjugated multiple copies of peptide antigen based on group A streptococcus M protein and human papillomavirus-16 E7 peptide to polymer cores in two separate experiments [31,42,44,82]. The reaction was performed in DMF, using copper wire as the Cu^l^ source (as a result of comproportion reaction of copper ions) and heating at 50 °C overnight [25,83]. Based on elemental analysis of the resulting products, the efficiency of the CuAAC reaction was relatively good with a substitution ratio of around 75% (Figure 8C) [31,42,44,82]. The product self-assembled into particles which induced strong and antigen-specific cellular [44] and humoral immune responses [31,42,82].

**Figure 9:**
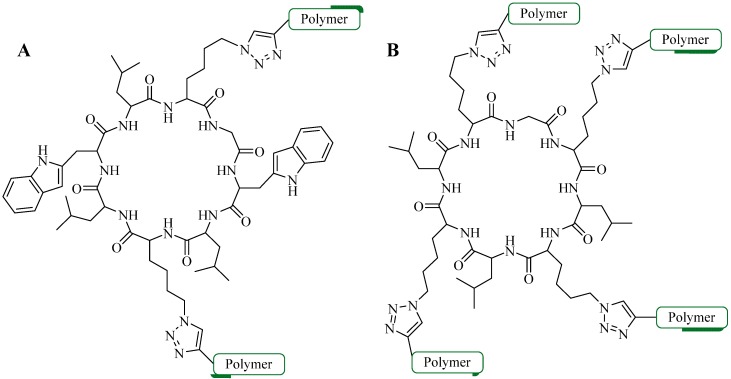
Cyclic peptides scaffold for CuAAC bioconjugation with PBA polymer: (**A**) two-arm cyclic peptide, (**B**) four-arm cyclic peptide.

Perrier and colleagues examined CuAAC conjugation efficiency between a cyclic β-sheet forming peptide and poly(butyl)acrylate (PBA) polymer [84]. The group observed steric effects that limited the reaction efficiency [84]. The conjugation was carried out using two- and four-arm cyclic functionalized peptides as illustrated in Figure 9. CuAAC reaction was performed using CuSO_4_, NaAsc in either: DMF, trifluoroethanol (TFE), hexafluoroisopropanol (HFIP), TFE/DMF mixture, or HFIP/DMF mixture, and irradiated under microwave at 100 °C. Conjugation of polymers with high degree of grafting and polymerization (DP = 108) with a four-arm peptide showed a maximum of 55% conjugation efficiency. However, quantitative efficiency was reported using lower DP polymers (16 and 36) [84]. Reduced coupling efficiency observed by Skwarczynski *et al*. during the polymer-peptide conjugation may also result from steric effects associated with the large hydrophobic polymeric block [31,44].

**Figure 10:**
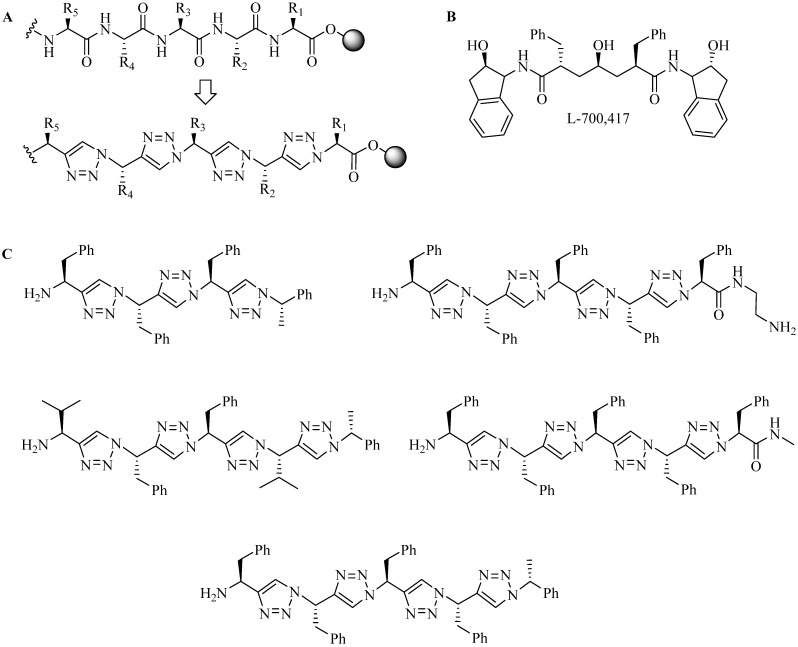
(**A**) Amide backbone modification with triazole rings; (**B**) HIVPR inhibitor; (**C**) Triazolamer-based HIVPR inhibitors.

Arora and co-workers used triazoles as substitutes for native peptide backbone (triazolamer) [85,86]. A fully triazole-based peptide backbone was synthesized by the group after careful optimization of the reaction (Figure 10A) [85,86]. The group exploited one-pot triazole synthesis via sequential zinc (II) catalyzed diazoltransfer reaction and CuAAC reaction on solid support (PAM resin). The α-amino group of the amino acid was substituted with an azide moiety using amino acid methyl ester, trifluoromethanesulfonyl azide (triflic azide), CuSO_4_, and triethylamine at room temperature for three hours. The CuAAC reaction was performed by the addition of alkyne-derivatised α-amino acid, TBTA ligand and NaAsc to the initial reaction mixture and stirring at room temperature for 18 h. Upon repetition of above process the final product was obtained with an overall yield of 78% [85]. Interestingly, NMR analysis of the triazolamer suggested that the peptidomimic adopt β-strand-like structures, although the structural backbone lacks β-strand’s hydrogen bond functionality [87]. The group further synthesized and evaluated triazolamer as human immunodeficiency virus-1 protease (HIVPR) inhibitor by synthesizing triazolamers that superimposed L-700,417 (Figure 10B), a peptide-based inhibitor that is widely used for HIVPR inhibition studies [87]. Five compounds (Figure 10C) showed high binding affinity (IC_50_ in micromolar range) compared to L-700,417 (IC_50_ = 670 µM) illustrating the viability of the triazolamers as peptidomimatic inhibitor.

**Figure 11:**
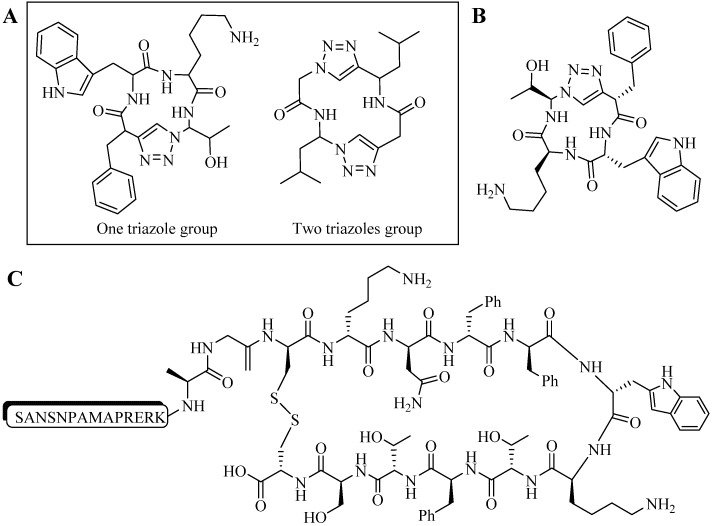
(**A**) General chemical structures of one and two triazole constructs. (**B**) Compound exhibited best somatostatin receptor binding experiment (IC_50_). (**C**) Chemical structure of SRIF-28.

Ghadiri and co-workers synthesized heterocyclic pseudotetrapeptide via CuAAC reactions. Mimicking small β-turn molecules, the constructs were used as probes to assess the conformation of ligands to target the somatostatin receptor [88]. These 13- or 14-membered ring constructs each bore one or two triazole rings as peptide backbone surrogates (Figure 11A) [88]. Initially, linear peptides were synthesized in solution phase and later were subjected to CuAAC reaction (CuI, 2,6-lutidine, DIPEA, TBTA in MeCN, stirred at room temperature for 12 h) to produce their cyclic counterparts. For the initial experiment, Ghandiri and co-workers synthesized a library of 16 cyclic isomeric compounds containing one triazole moiety. HPLC yields of 31% to 90% were obtained. Additionally, binding affinity experiments were carried out for the library. Although one compound showed high binding affinity (IC_50_ < 200 nM, Figure 11B), its affinity was lower than the parent peptide, SRIF-28 (IC_50_ < 5 nM, Figure 11C). Cyclic tetrapeptides without triazol moieties were not tested as the synthesis of the 12-membered ring resulted in very poor yield [88].

**Figure 12:**
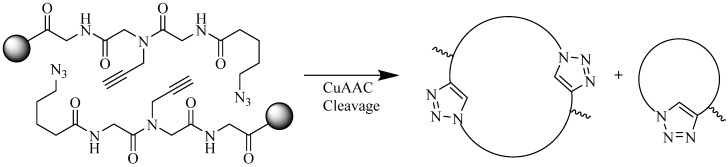
Head-to-tail CuAAC conjugation producing cyclodimer or cyclomonomer.

Jagasia *et al*. used CuAAC as a means to form a head-to-tail cyclodimerized construct on resin (with intact side-chain protecting groups, Figure 12). The group investigated some properties that could influence the formation of the cyclodimer pseudopeptide, as oppose to a monocyclic pseudopeptide which was mainly the aimed for some researchers [72,73,79,89,90]. Each CuAAC reaction was performed using CuI, DMSO:MeCN (1:3), at room temperature for 40 h [89]. It was concluded that cyclization of the peptide via CuAAC can be influenced by:

(a) The distance between the active groups (azide or alkyne) and the resin—as the distance increased, the yield of bicyclic product decreased;
(b) The distance between the active groups—a minimum of six amino acids promotes cyclodimerization;
(c) Solvent composition ratio—affects resin swelling and interstrand hydrogen bonding thus affecting the dimerization, and;
(d) The structural homolog of the peptide (α, β, γ *etc*.)—α and β homologs readily form the cyclodimer while γ homologs do not [89].

**Figure 13:**
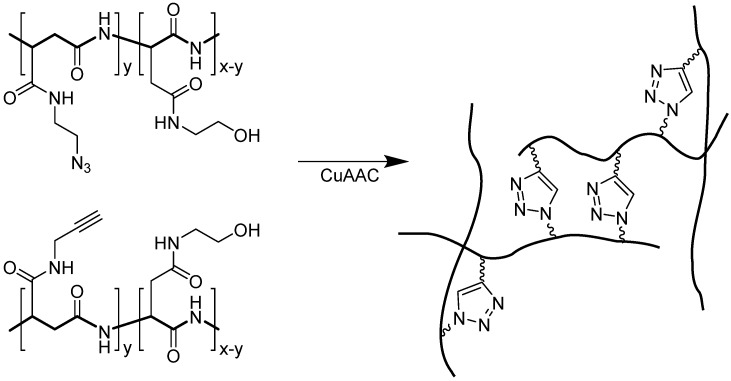
Formation of hydrogel based on multilinker conjugation via CuAAC.

An example of crosslinking using CuAAC is illustrated in Figure 13. Kim and coworkers used polyaspartimide derivatives to construct a biocompatible, biodegradable three-dimensional hydrogel network that was cross-linked via the CuAAC reaction [91]. The reaction was performed using CuBr in the presence of *N*,*N*,*N’*,*N’*,*N’’*-pentamethylenetriamine ligand. The polymer hydrogel was reported to form within minutes [91].

### 4.2. Intramolecular Triazoles Linker

#### 4.2.1. Side Chain Stapling/Macrocyclization

Peptides and proteins fold in a well-defined conformation in order to maintain their biological activity. Short peptides, such as APR-1 and J14_i_ epitopes, have very distinct well-known drawbacks including susceptibility to protease degradation, poor bioavailability, and a highly flexible structure [43,92]. When used as vaccines, these peptides were poorly recognized by the immune system, resulting in weak antibody production. One of possible methods to improve the conformational stability of a peptide is by flanking it with helix promoting sequences, (e.g., GCN4 sequences) [92,93]. Alternatively, a side-chain stapling method can be applied. This method introduces conformational constraints in peptides via side-chain-to-side-chain conjugation to stabilize peptide conformation and further improving peptides’ stability against enzymatic degradation [56].

Previous, structural studies of 3_10_ helix based on a 4-aminopiperidine-4-carboxylic acid (Api) and α-aminoisobutyric acid (Aib) rich peptide was performed by Ousaka *et al*. Efforts to use a ‘locked’ 3_10_ helix approach via olefinic bridge and *p*-phenylenediacetic acid bridge were unsuccessful due to distortion of the native helix structure caused by the hydrophobic moiety of the olefinic and phenylenediacetic acid linkers [94,95]. Klaveness and co-workers used the CuAAC reaction to perform a novel stapling strategy with a Aib-rich model peptide [56]. The CuAAC stapling strategy was preferable because it established a hydrophilic 1,2,3-triazole moiety in the linker [56]. The model peptides were cyclized using CuI, P(OEt)_3_, DIPEA in CH_2_Cl_2_ at room temperature for 42 h. The CuAAC reaction produced the desired intramoleculary-linked peptide in 83% yield. Crystallographic analysis of the constructs confirmed formation of a nearly perfect 3_10_ helical peptide (angle σ^2^ < 2^o^) [56].

**Figure 14:**
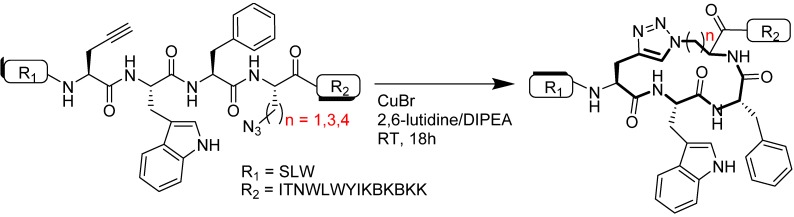
3_10_ helix side-chain-to-side-chain CuAAC cyclization.

Dawson and Ingale used side-chain-to-side-chain CuAAC conjugation to construct a modified HIV-1 gp41 peptide structure (SLW**J**WF**K(N_3_)**ITNWLWYIK**Aib**K**Aib**KK, where **J** is propargylglycine, Pra) as illustrated in Figure 14. The side chain conjugation was performed between **J** and azidolysine, **K(N_3_)**, located at 3_10_ helix position in order to stabilize the kink formed by the tryptophan and phenylalanine amino acids [90]. Dawson and Ingale initially faced difficulties during the cyclization using CuI, 2,6-lutidine in DMSO or DMF, even after a prolonged period of time. However, changing the reaction system to a CuBr/2,6-lutidine/DIPEA mixture resulted in 70% HPLC yield of product after 18 h. Analysis of the construct via circular dichroism showed the highest helicity content (based on mean residue ellipticity) for macrocycles with a 17- or 18- membered ring compared to their linear or 15 membered ring counterparts [90]. In comparison to the 3_10_ helix system, optimization in α-helix system performed by D’Ursi *et al*. illustrated that the best helical stabilization was achieved for constructs that possessed a 19 membered side-chain to side chain linkage [96].

**Figure 15:**
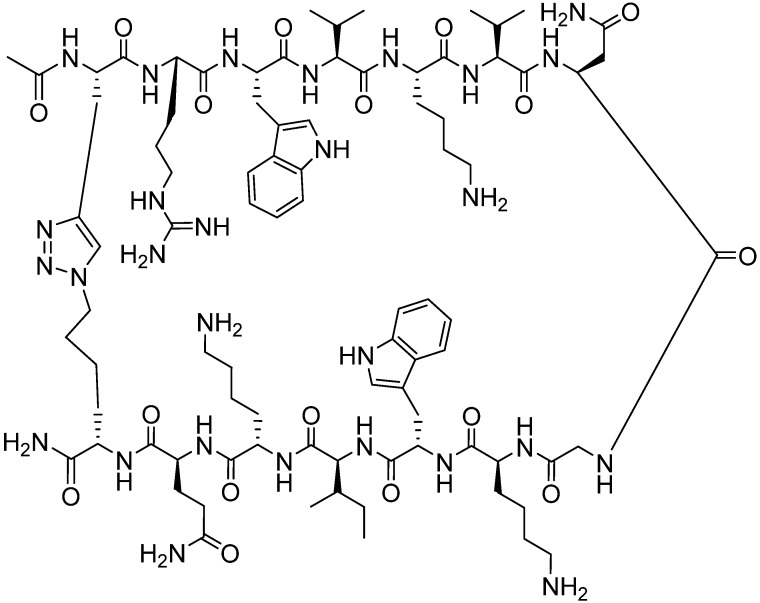
Structural improvement via side chain triazole linker.

More recently, Waters and Park used the CuAAC reaction to investigate the effect of cyclization on the structure, stability and activity of a β-hairpin tryptophan-lysine-tryptophan-lysine, WKWK peptide [97]. The wild type (wt) peptide was shown to be able to form β-hairpin structure, had low stability under protease treatment (three minutes), and was able to bind to adenosine triphosphate (ATP) nucleic acid [97]. The WKWK peptide was modified by placing azido and alkyl groups at several positions within the structure [98]. CuAAC reaction was performed in phosphate buffer (10 mM, pH 8), in the presence of tris-tri(methylazolyl) amine ligand, NaAsc, and tetrakis(acetonitrile)copper(I)hexaflurophosphate at room temperature overnight. It was found that triazole linkage of the C-terminal side-chain to the N-terminal side-chain (Figure 15) maintained the β-structure more readily than the wt hairpin. Cyclization through the triazole linkage afforded improved proteolytic (>10 fold) and thermal stability. The ability of the macrocyclized construct to bind to ATP was better than its wt counterpart, with IC_50_ of 110 µM and 179 µM, respectively [97].

#### 4.2.2. Triazoles as Disulfide Bridge Substitutions

**Figure 16:**
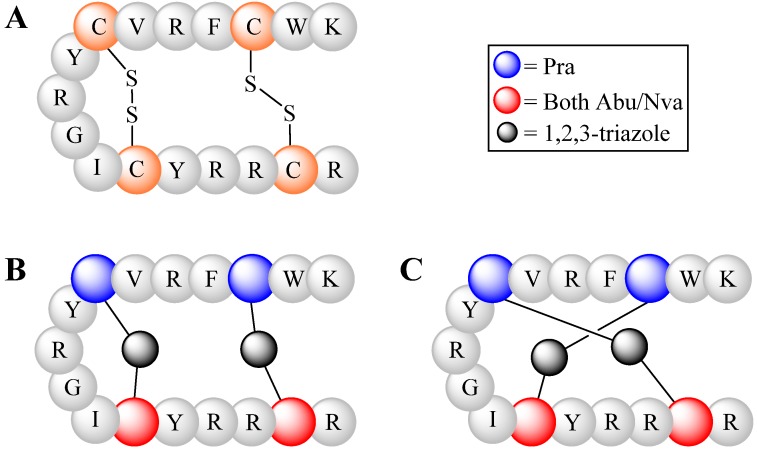
β-Hairpin structure of TP-1 peptide (in single letter amino acid code). (**A**) Native structure with two disulfide bonds. (**B**) Hairpin-like structure via triazole linkers. (**C**) Globule-like structure due to incorrect folding.

Disulfide bonds (bridges) play an important role in the integrity of folded peptides and proteins. For example, a 17 amino acid long antimicrobial peptide tachyplesin I (TP-1), contains two disulfide bridges that are crucial for correct β-hairpin folding and therefore its activity. Holland-Nell and Meldal used the CuAAC approach to substitute these two bridges with triazoles linkers by replacing two cysteines with Pra and the other two cysteines with either 2-amino-4-azidobutyric acid (Abu) or 5-azidonorvaline (Nva) [99]. They successfully formed correctly folded β-hairpin analogs, but the majority of the product was in the form of a misfolded globule-like structure (Figure 16, **B**:**C** in 1:7 ratio), following a CuAAC reaction using CuSO_4_/tris(carboxyethyl)phosphine in H_2_O for 16 h on resin. Microwave assisted CuAAC shifted the triazole formation, favoring the hairpin structure (**B**:**C** in 1:1.5 ratio). It was suggested that CuAAC reaction in an aqueous environment favored intramolecular hairpin folding while minimizing interchain bonding, as oligomerization was not observed at both conditions. Inhibition experiment (MIC) of the triazole analogs showed improved antibiotic activity against some bacterial species (*Escherichia coli*, *Bacillus subtilis* and *Salmonella typhimurium*) compared to wild type TP-1 peptide.

### 4.3. Other Applications

**Figure 17:**
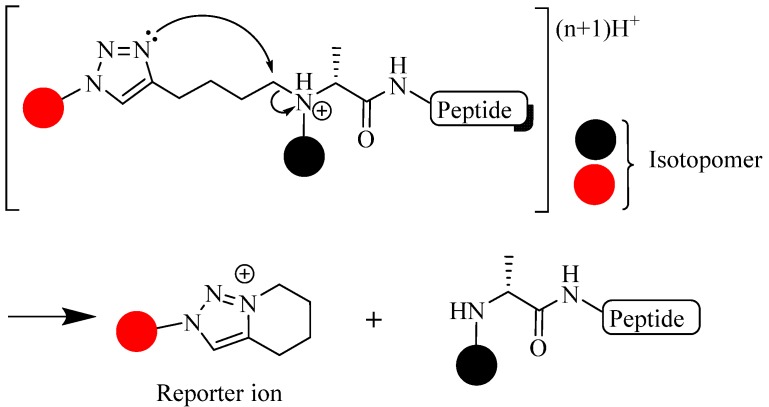
Gas phase fragmentation of triazole into reporter ion.

A unique application of the CuAAC reaction involves the use of a triazole moiety as a gas-phase cleavable linker for protein/peptide quantification under mass spectroscopy (MS)-ionization condition [100]. Although triazole is known for its stability as peptide/protein linker [101], Sohn *et al*. showed that it could be cleaved during MS measurement and form ionized species (reporter ion) which, in turn, was easily recorded by an ion detector (Figure 17). The CuAAC reaction to produced labeled peptide was performed using; CuSO_4_, NaAsc, TBTA, in DMSO/H_2_O mixture, at room temperature for 4 h (yield = 69%–72%) [100].

## 5. Conclusions

The structural conformation of peptides and proteins are crucial for their biological activity. Chemical conjugation of biomolecules and amide-to-triazole substitutions via copper (I) catalyzed alkyne azide 1,3-dipolar cycloadditions (CuAACs) were shown to have the potential for improvement in medical applications such as, but not limited to, tumor-targeting/tumor-detecting ability as well as the potential for improved drug stability and efficacy. These limitless capacities of CuAAC result from the selectivity of the reaction, ease to perform, and various choice of medium. To further exploit this reaction, detailed investigation into CuAAC transition states should be carried out to determine the ‘true’ reaction mechanism and therefore achieve better control of the reaction itself. Although CuAAC was shown to be a very robust reaction and is widely used in peptide chemistry, its application in protein chemistry is therefore possible. There are many opportunities for the expansion of CuAAC into the field of protein modification and beyond.
